# Functional proprioceptive stimulation promotes early rehabilitation following ACL reconstruction: a randomized controlled trial

**DOI:** 10.3389/fresc.2026.1882498

**Published:** 2026-07-16

**Authors:** Hervé Collado, Pierre-Yves Tourtel, Thomas Bardot, Thibaut Fraysse, Emma Paul, Marion Prats, Frédéric Chorin, Serge S. Colson

**Affiliations:** 1 Clinique Saint-Martin Sport, Marseille, France; 2 Clinique Saint-Martin Sud, Marseille, France; 3 Université Côte d’Azur, CHU, Nice, France; 4 Université Côte d’Azur, LAMHESS, Nice, France

**Keywords:** ACL reconstruction, arthrogenic muscle inhibition, continuous passive motion, kinesthetic illusion, local vibration, pain, sensorimotor rehabilitation

## Abstract

**Introduction:**

Early-phase rehabilitation following anterior cruciate ligament reconstruction (ACLR) should prioritize the recovery of knee joint mobility while considering sensorimotor alterations that may occur after injury and surgery. Continuous passive motion (CPM) and functional proprioceptive stimulation (FPS) may support joint mobility recovery, but their combined effects during early rehabilitation after ACLR have not yet been investigated. This preliminary randomized controlled trial evaluated the effects of combining FPS with CPM compared with CPM alone during the first two weeks of rehabilitation following ACLR.

**Methods:**

Twenty-four participants undergoing ACLR were randomly allocated to either a CPM + FPS group or a CPM group. Participants completed a standardized physiotherapy program consisting of 10 sessions over 2 weeks. Depending on group allocation, participants received CPM alone or CPM combined with synchronized FPS during the first 30 min of each session. Active range of motion (AROM), passive range of motion (PROM), active and passive knee extension deficit, pain intensity, and daily analgesic use were assessed throughout the intervention. Repeated continuous outcomes were analyzed using linear mixed-effects models including participant random intercepts, while analgesic use was analyzed as a repeated binary outcome using a binomial mixed-effects model.

**Results:**

A significant group × time interaction was observed for AROM (*p* = 0.001), with greater improvement in the CPM + FPS group than in the CPM group (+48.6° vs. +31.4°, *p* = 0.036). Although the CPM + FPS group demonstrated a larger post-intervention PROM gain than the CPM group (+43.5° vs. +31.1°), this was not significant (*p* = 0.083). Pain intensity (*p* < 0.001) and analgesic medication (*p* < 0.05) decreased over the 2-week period without significant between-group difference.

**Discussion:**

Within a 2-week postoperative standardized early rehabilitation program following ACLR, the addition of FPS to CPM was associated with greater improvement in AROM. Although PROM also tended to improve to a greater extent in the CPM + FPS group, the evidence supporting this finding was less robust. No significant between-group difference was observed for pain intensity. These preliminary findings support the potential value of FPS as a pragmatic adjunct to CPM for early mobility recovery.

**Clinical Trial Registration**: [ClinicalTrials.gov], identifier NCT07553013.

## 1 Introduction

Anterior cruciate ligament (ACL) injury is one of the most common knee injuries in physically active populations and frequently results in prolonged functional limitations and reduced participation in sports activities. Depending on the degree of instability, activity demands, associated injuries, and patient characteristics, management may involve either structured rehabilitation alone or anterior cruciate ligament reconstruction (ACLR) followed by rehabilitation (1). In addition to restoring biomechanical stability and functional capacity, growing evidence suggests that ACL injury and reconstruction may also be associated with sensorimotor and neurophysiological alterations affecting movement control and proprioceptive function (2, 3). Following ACL injury, changes in afferent signaling arising from damaged mechanoreceptors, joint swelling, inflammation, and pain may alter somatosensory feedback from the knee joint (4). Furthermore, alterations in spinal reflex activity and corticospinal excitability have been reported after ACL injury and reconstruction, although the precise mechanisms and their clinical implications remain under investigation (5). Together, these alterations have been proposed as contributors to arthrogenic muscle inhibition (AMI) and persistent sensorimotor deficits observed in some individuals following ACL injury and reconstruction (6, 7). Therefore, therapeutic strategies designed to enhance sensorimotor function and proprioceptive input may represent useful adjuncts to conventional rehabilitation approaches, particularly during the acute/early postoperative phase, approximately the first 2–4 weeks after ACL reconstruction (3, 8, 9).

Functional proprioceptive stimulation (FPS) represents a promising intervention for enhancing peripheral-central sensorimotor interactions (10–12). FPS consists of local vibrations applied to the musculotendinous junction of a relaxed muscle, which activate muscle spindle afferents and provide the central nervous system with information about changes in muscle length (10, 11). Even in the absence of actual limb movement, FPS can induce a vibration-induced kinesthetic illusion of movement, as the activation of muscle spindles mimics the sensory signal generated during muscle stretching (11). Proprio-tactile stimulation based on vibration sequences mimicking natural movements has been shown to preserve sensorimotor network integrity and maintain movement amplitudes during periods of immobilization (13). In the context of ACL rehabilitation, FPS may therefore provide additional proprioceptive input following ligament injury, although its specific neurophysiological effects after ACLR remain to be established (12–14). Vibration may also contribute to pain modulation through non-nociceptive sensory input, as proposed by the gate control theory (15), and experimental work suggests that vibration-induced kinesthetic illusions may influence pain perception (16). However, pain should not be interpreted simplistically during physiotherapy, as it can also guide load management and tissue protection.

During the early postoperative phase of ACL rehabilitation (approximately the first 2–4 weeks after surgery, and particularly during the first 2 postoperative weeks), restoring knee joint mobility through pain-free exercises is one of the primary objectives (17, 18). Continuous passive motion (CPM) devices have been used to facilitate early joint mobilization following ACLR. Early studies suggested that CPM could improve range of motion and reduce postoperative pain compared with immobilization or active movement alone (19, 20). However, subsequent studies reported conflicting results, and several systematic reviews concluded that the clinical benefits of CPM remain uncertain (21, 22). Consequently, some clinical practice guidelines discourage the routine use of CPM following ACLR (23, 24). Nevertheless, another recent systematic review with meta-analyses reported beneficial effects of CPM on early knee flexion recovery and pain reduction (25). In addition, clinical practice guidelines (26) and current practices (27) still recommend the use of CPM during the early postoperative period. Another important objective during the early phase is to reduce spinal reflex inhibition and enhance knee extensor muscle activation affected by AMI (8). In this regard, reviews have suggested local vibration among possible interventions after ACLR (28, 29).

Previous studies investigating local vibration after ACL reconstruction remain limited and have mainly focused on neuromuscular outcomes. Local vibration has been shown to acutely improve quadriceps function and muscle activation (30), while repeated vibration sessions during rehabilitation have been associated with enhanced quadriceps strength recovery (31). Although these findings support the potential of vibration-based interventions after ACLR, their effects on early postoperative knee mobility, pain-related outcomes, and sensorimotor recovery remain poorly documented. Moreover, the combination of FPS with CPM has not previously been investigated.

One of the earliest published investigations of functional proprioceptive stimulation was reported in the early 1980s by Neiger et al. (32). Although historical, this study suggested that FPS may facilitate joint mobility recovery following immobilization and provided the basis for subsequent developments in vibration-based sensorimotor rehabilitation. More recently, daily application of FPS on the contralateral limb during the first postoperative week after wrist surgery was associated with reduced pain perception and improved range of motion during follow-up compared with a control group (33). Additionally, a combined intervention including CPM, local vibration, and thermotherapy has been shown to improve joint mobility and reduce pain in patients with knee osteoarthritis compared with a sham intervention (34). Taken together, these observations suggest that CPM and FPS may both contribute to joint mobility recovery after immobilization or surgery. However, the combined effects of FPS and CPM during the early phase of rehabilitation after ACLR have not yet been investigated. Furthermore, although internal tibial rotation and multiplanar joint control are key components of ACL injury mechanisms, the present study was designed to focus on early postoperative sagittal-plane mobility and pain-related outcomes, which represent the primary targets of CPM-based rehabilitation during this phase.

Therefore, the purpose of the present preliminary randomized controlled trial was to assess the efficacy of a rehabilitation intervention combining CPM and FPS during the early postoperative phase following ACL reconstruction (i.e., within the first 2 weeks). The primary outcome was active knee range of motion recovery. We hypothesized that the combination of CPM and FPS would be associated with greater improvements in active knee range of motion compared with CPM alone. PROM, knee extension deficit, pain intensity, and analgesic medication use were considered secondary outcomes.

## 2 Materials and methods

### 2.1 Participants

**Figure 1:**
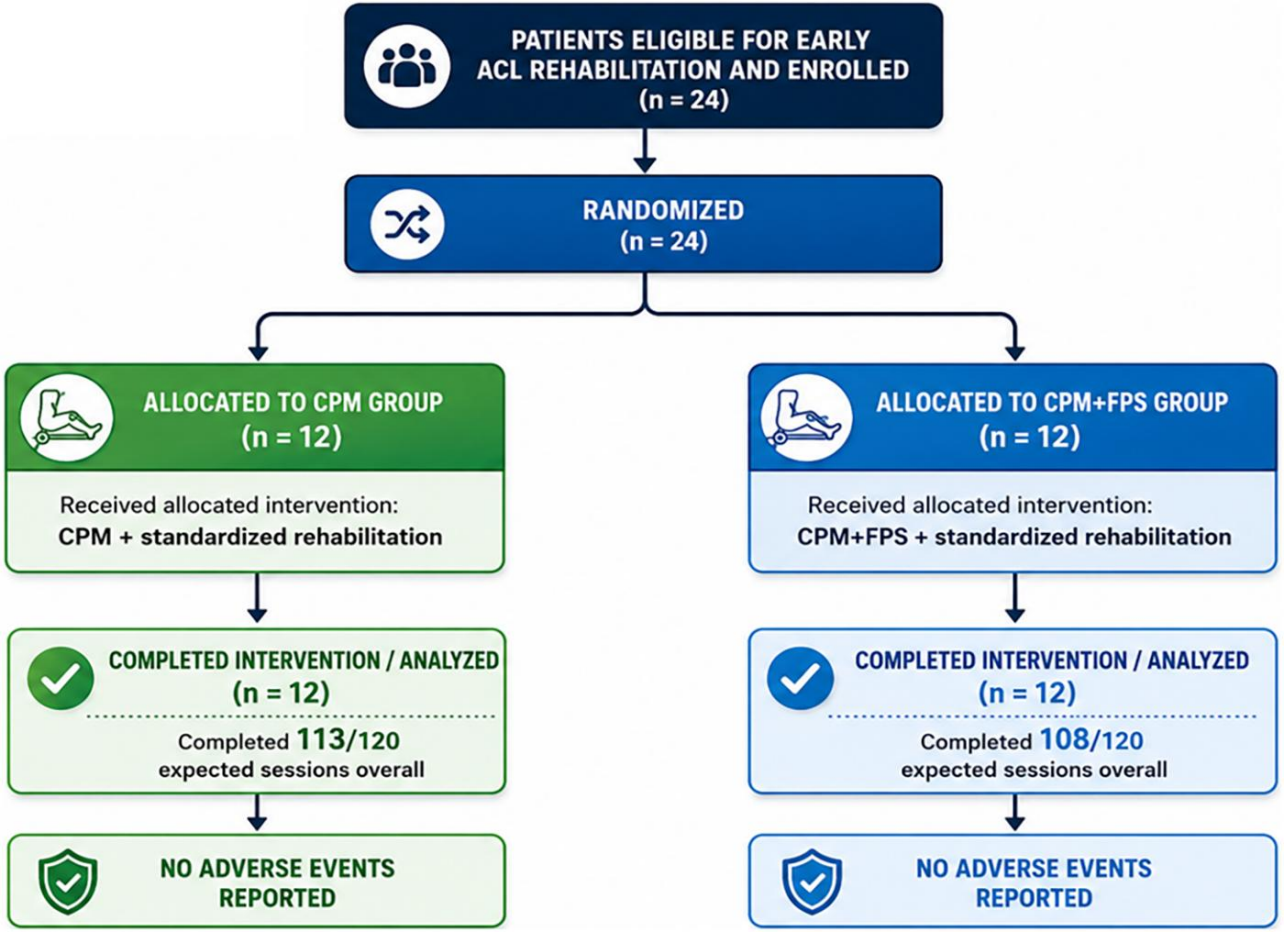
CONSORT-style participant flow diagram showing available information on enrollment, randomization, allocation, follow-up, analysis, adherence, and adverse events for the two rehabilitation groups. Detailed screening logs were not prospectively maintained; therefore, the numbers of all screened, excluded, or declining participants could not be reconstructed retrospectively.

The required sample size was estimated *a priori* using G*Power (version 3.1.9.6; Kiel University, Kiel, Germany). The primary outcome was active knee range of motion (AROM) recovery. The calculation was based on data from Neiger et al. (32), one of the few published studies examining joint mobility recovery following FPS. A repeated-measures design with a within-between interaction was assumed, with an alpha level of 0.05 and statistical power of 0.95. The resulting calculation indicated a minimum total sample size of eight participants. Because the present study represented the first randomized trial evaluating the combination of CPM and FPS after ACL reconstruction, 24 participants were ultimately recruited (allocation ratio 1:1) to improve robustness, reduce the influence of interindividual variability, and limit the impact of potential missing data or participant withdrawal. Participants were consecutively recruited among patients scheduled for ACL rehabilitation at the Clinique Saint-Martin Sport (Marseille, France). Twenty-four participants provided written informed consent before participation (age: 29.9 ± 8.6 years; mean ± standard deviation). A sensitivity power analysis indicated that the final sample size of participants included in the present study (*n* = 24) allowed detection of a medium effect size (f = 0.39) with alpha = 0.05 and power = 0.95. All experimental procedures were conducted in accordance with the Declaration of Helsinki (2013). The study protocol was approved by the Comité d'Evaluation de la Recherche STAPS (IRB00012476-2025-04-04-397) and registered at ClinicalTrials.gov (NCT07553013). The participant flow is presented in Figure 1. The trial is reported in accordance with CONSORT recommendations.

Participants were eligible if they met the following inclusion criteria: male or female aged 18–55 years, isolated ACL rupture reconstructed using a four-stranded semitendinosus autograft, with or without associated extra-articular tenodesis, and enrollment in the rehabilitation program between the fourth and seventh postoperative day.

Exclusion criteria were: previous ligament reconstruction of the involved knee, reconstruction using a patellar tendon (bone-patellar tendon-bone) autograft or quadriceps tendon autograft, presence of major neurological or musculoskeletal disorders (e.g., multiple trauma, Parkinson's disease, cerebral palsy, stroke, Alzheimer's disease, dementia), cognitive impairment (i.e., Mini Mental State Examination score <18), and body mass index greater than 35 kg/m^2^.

### 2.2 Randomization and rehabilitation intervention

The study was conducted over a two-week rehabilitation period. Clinical examination and assessment of knee joint mobility were performed before and after the rehabilitation program, while pain was monitored throughout the intervention. Participants were randomly allocated in a 1:1 ratio to either the CPM group or the CPM + FPS group. The randomization sequence was computer-generated before the start of recruitment by a member of the research team who was not involved in participant enrolment, treatment delivery, or outcome assessment. Allocation concealment was ensured using sequentially numbered, opaque, sealed envelopes. After eligibility confirmation, written informed consent, and baseline assessment, the next envelope in the sequence was opened to determine group assignment. Participants were enrolled by the clinical team, and group allocation was communicated only to the physiotherapists responsible for delivering the intervention. Outcome assessments were performed by the same examiner, who was not involved in treatment delivery and remained blind to group allocation throughout the study. Participants were instructed not to disclose their allocation during assessments. Due to the nature of the intervention, neither participants nor physiotherapists could be blinded because the vibratory stimulation delivered by the FPS device was readily perceptible. Participants were asked to maintain their usual daily life habits outside the clinic during the study period.

**Figure 2:**
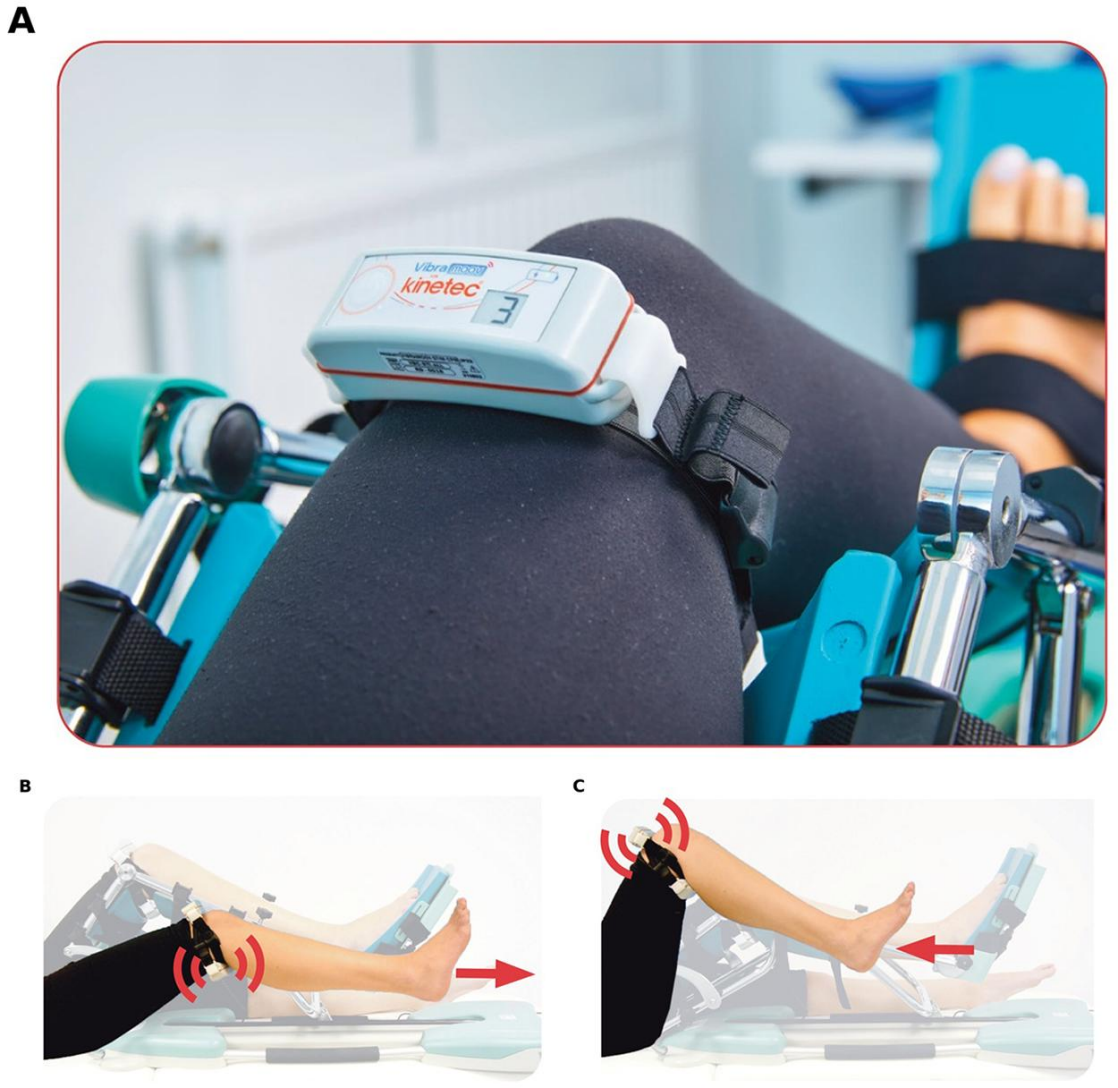
CPM + FPS rehabilitation setup and intended movement perceptions. **(A)** Illustration of a CPM + FPS rehabilitation session. The lower limb was placed in an arthromotor, and two stimulators were positioned at the distal musculotendinous junction of the quadriceps and at the popliteal fossa. **(B)** Popliteal fossa stimulation was intended to induce a perception of extension greater than that conveyed by CPM. **(C)** Quadriceps stimulation was intended to induce a perception of flexion greater than that conveyed by CPM.

Ten rehabilitation sessions were performed over 2 consecutive weeks (Monday to Friday). Each session lasted three hours and followed a standardized physiotherapy protocol applied identically in both groups, except for the experimental condition delivered during the first 30 min. Each session consisted of 5 stages: Stage 1 (30 min): CPM or CPM + FPS depending on group allocation. Stage 2 (60 min): muscle activation of the quadriceps and hamstrings with isometric contractions, squats (with technical assistance if needed), leg press, glute bridges, and work of the gluteus medius and gastrocnemius, mobility, and strengthening exercises (including knee extensor activation, closed-chain exercises, and controlled range-of-motion tasks performed within a pain-free range). Stage 3 (30 min): gait training using an anti-gravity treadmill (AlterG Pro, AlterG Inc., Fremont, CA, USA), including progressive weight-bearing and coordination tasks. Stage 4 (30 min): core stability and upper-limb strengthening exercises contributing to postural control and neuromuscular coordination. Stage 5 (30 min): manual physiotherapy techniques (including joint mobilization, soft tissue techniques, manual lymphatic drainage massage and passive stretching when appropriate) followed by compressive cryotherapy and pressotherapy. To ensure intervention fidelity, all rehabilitation sessions followed a predefined standardized protocol implemented by physiotherapists from the same clinical team. Each physiotherapist treated participants from both groups and applied identical rehabilitation procedures outside the allocated intervention. Progression of CPM amplitudes was individualized according to the maximal pain-free range of motion tolerated by each participant. The content, duration, and sequence of the remaining rehabilitation stages were identical for both groups throughout the study period. During the first stage, CPM exercises were performed using an arthromotor device (Kinetec Spectra Essential™, Kinetec SAS, Tournes, France). Knee flexion and extension amplitudes were initially set by the clinician at the maximal pain-free range of motion for each participant. During the session, participants could increase flexion or extension amplitudes using a remote control provided that no pain was experienced. In the CPM + FPS group, FPS was delivered synchronously with the passive movements generated by the CPM device. Two stimulators (Vibramoov STIM CPM, Techno Concept, Manosque, France) were positioned at the distal musculotendinous junction of the quadriceps (above the patella) and at the popliteal fossa. These locations were selected to stimulate muscle spindle afferents involved in knee flexion-extension sensorimotor signaling, with quadriceps stimulation intended to induce a perception of flexion and popliteal stimulation intended to induce a perception of extension. The vibratory stimulation was delivered continuously during joint movement (Figure 2). Vibration frequencies ranged from 40 to 100 Hz and were automatically adjusted according to the speed and amplitude of the CPM movement. Frequency modulation was controlled by an embedded algorithm using inertial sensors (accelerometers and gyrometers) to synchronize stimulation with CPM movement in real time, ensuring reproducibility and consistency across sessions. Participants were monitored throughout the rehabilitation program by the clinical team. Adverse events were defined as any unexpected medical event occurring during the study period that required modification, interruption, or discontinuation of rehabilitation or additional medical evaluation. Examples included excessive pain, clinically significant swelling, wound complications, infection, falls, or any event judged by the treating clinician to interfere with rehabilitation.

### 2.3 Clinical outcomes

Before the two-week period, all participants underwent a medical examination and clinical assessments. Baseline demographic, clinical, and surgical characteristics were recorded before rehabilitation initiation when available. These included age, sex, time from surgery to rehabilitation initiation, baseline functional status, baseline range of motion, and baseline pain measures. Functional limitations were assessed using the French version of the Knee Injury and Osteoarthritis Outcome Score—Physical Function Short form (KOOS-PS) (35). The KOOS-PS includes seven items assessing common functional activities and is scored on a five-point Likert scale ranging from 0 (no difficulty) to 4 (extreme difficulty). KOOS-PS was used to characterize baseline functional status and was not repeated during the short intervention period.

The maximal pain-free amplitudes of knee flexion in passive (PROM) and active (AROM) positions were measured with a 30-cm-long manual goniometer (36) at the end of Stage 1 during the first (PRE), fifth (MID), and tenth (POST) rehabilitation sessions. Similarly, maximal pain-free amplitudes of passive and active knee extension deficit were collected with the goniometer. All measurements were performed by the same examiner, who was not involved in the physiotherapy sessions and was blinded to group allocation. A standardized assessment protocol was used with participants positioned supine with the pelvis aligned, the hip neutral, and care taken to avoid compensatory movements. AROM was assessed using a consistent procedure across sessions. In addition, the passive ranges of motion of the arthromotors were recorded at the beginning and at the end of each session.

Pain intensity was assessed using a visual analogue scale (VAS) ranging from 0 (no pain) to 10 (worst imaginable pain). Pain scores were recorded at the beginning and at the end of each rehabilitation session. Participants reported whether analgesic medication had been used on each rehabilitation day. Medication use was recorded as a binary variable (use vs. non-use). No study-specific medication protocol was imposed, and postoperative medication management remained under the responsibility of the treating physicians according to routine clinical practice. Information regarding medication type, dosage, timing of administration, and use of anti-inflammatory drugs was not systematically collected.

### 2.4 Statistical analysis

Statistical analyses were performed using IBM SPSS Statistics for Windows (version 26, IBM Corp., Armonk, NY, USA). The normality of data distribution was assessed using the Shapiro–Wilk test. Baseline characteristics between groups were compared using one-way ANOVA or Kruskal–Wallis tests depending on variable type and data distribution. For all repeated continuous outcomes, participant was included as a random intercept to account for within-participant clustering. AROM, PROM, and active and passive knee extension deficit were analyzed using linear mixed-effects models with normal residual distribution and identity link function. These models included group (CPM vs. CPM + FPS), time (PRE, MID, POST), and the group × time interaction as fixed effects. Pain scores and arthromotor amplitudes were analyzed using linear mixed-effects models including group, session (1–10), time within session (before vs. after session), and their interactions as fixed effects, with participant included as a random intercept. Analgesic medication intake was analyzed as a repeated binary outcome using a generalized linear mixed-effects model with a binomial distribution and logit link function. This model included group, session, and the group × session interaction as fixed effects, with participant included as a random intercept. Missing observations resulting from missed rehabilitation sessions or early discharge were handled within the mixed-effects modelling framework. Mixed models use all available observations and accommodate unbalanced repeated-measures data under the assumption that data are missing at random. Participants were therefore not excluded solely because of incomplete attendance, and no imputation procedure was applied. Model convergence was checked for all models. For continuous outcomes, residual distributions were visually inspected to verify model assumptions. When models displayed significant interaction or main effects (*p* < 0.05), Bonferroni-adjusted pairwise comparisons were performed. Cohen's d effect sizes were computed to support interpretation of significant continuous outcomes (0.2 < small < 0.5; 0.5 ≤ medium < 0.8; large ≥ 0.8). Numerical data reported in the text and tables are expressed as mean ± standard deviation (SD) and 95% confidence intervals (95% CI) where appropriate. For graphical presentation, error bars are described individually in each figure caption and represent either SD or standard error (SE), as indicated.

## 3 Results

### 3.1 Participant characteristics

The CPM group included seven females and five males aged 19–44 years (29.8 ± 8.1 years), while the CPM + FPS group included six females and six males aged 18–50 years (30.0 ± 9.5 years). No significant difference was observed between groups for age (*p* = 0.964) or sex distribution (*p* = 0.68). Baseline KOOS-PS scores did not differ between groups (H(1, *N* = 23) = 0.598; *p* = 0.439), with values of 15.6 ± 6.5, 95% CI [11.5, 19.7] and 14.5 ± 4.8, 95% CI [11.5, 17.6] for CPM and CPM + FPS, respectively.

No significant between-group differences were observed regarding the time elapsed between surgery and the first rehabilitation session (*p* = 0.636) and the number of rehabilitation sessions completed (*p* = 0.730). Participants in the CPM group initiated rehabilitation at 5.8 ± 1.4 days after surgery and completed 9.4 ± 0.7 sessions (113/120 expected sessions; 94.2% adherence), whereas participants in the CPM + FPS group started rehabilitation 5.6 ± 1.6 days after surgery and completed 9.0 ± 1.9 sessions (108/120 expected sessions; 90.0% adherence). Baseline age, sex distribution, time elapsed between surgery and rehabilitation initiation, KOOS-PS scores, AROM, PROM, active and passive knee extension deficit are provided in Table 1. Most missing observations were attributable to occasional missed rehabilitation sessions related to minor scheduling constraints or early discharge following satisfactory clinical progression. No participant formally withdrew from the study after enrolment. No adverse events meeting the predefined study criteria were reported during the rehabilitation period in either group.

**Table 1:** Baseline characteristics of participants (upper panel) for both groups and knee joint mobility values (lower panel) measured at PRE (baseline), MID (after first week) and POST (at the end of the second week).

Participant characteristics at baseline				
	Group			
Age (years)	CPM	29.8 ± 8.1		
	CPM + FPS	30.0 ± 9.5		
Sex (Female/Male)	CPM	7/5		
	CPM + FPS	6/6		
KOOS-PS score	CPM	15.6 ± 6.5		
	CPM + FPS	14.5 ± 4.8		
Days between surgery and rehabilitation initiation	CPM	5.8 ± 1.4		
	CPM + FPS	5.6 ± 1.6		

Knee joint mobility				
	Group	PRE	MID	POST
AROM (°)	CPM	56.6 ± 10.2	78.4 ± 9.5	88.0 ± 8.4
	CPM + FPS	49.9 ± 15.9	85.0 ± 16.4	98.5 ± 9.2
PROM (°)	CPM	63.8 ± 11.8	86.1 ± 12.7	94.9 ± 8.3
	CPM + FPS	62.2 ± 15.1	90.6 ± 18.3	105.7 ± 10.9
Active knee extension deficit (°)	CPM	−6.8 ± 7.0	−3.9 ± 5.7	−2.5 ± 3.3
	CPM + FPS	−8.2 ± 7.0	−5.7 ± 6.9	−2.5 ± 5.0
Passive knee extension deficit (°)	CPM	−6.4 ± 6.6	−3.0 ± 4.8	−1.7 ± 2.5
	CPM + FPS	−6.2 ± 6.4	−4.4 ± 5.7	−2.5 ± 5.0

CPM, continuous passive motion. CPM + FPS, continuous passive motion + functional proprioceptive stimulation. AROM, active range of motion. PROM, passive range of motion. Values are mean ± SD.

### 3.2 Knee range of motion

**Figure 3:**
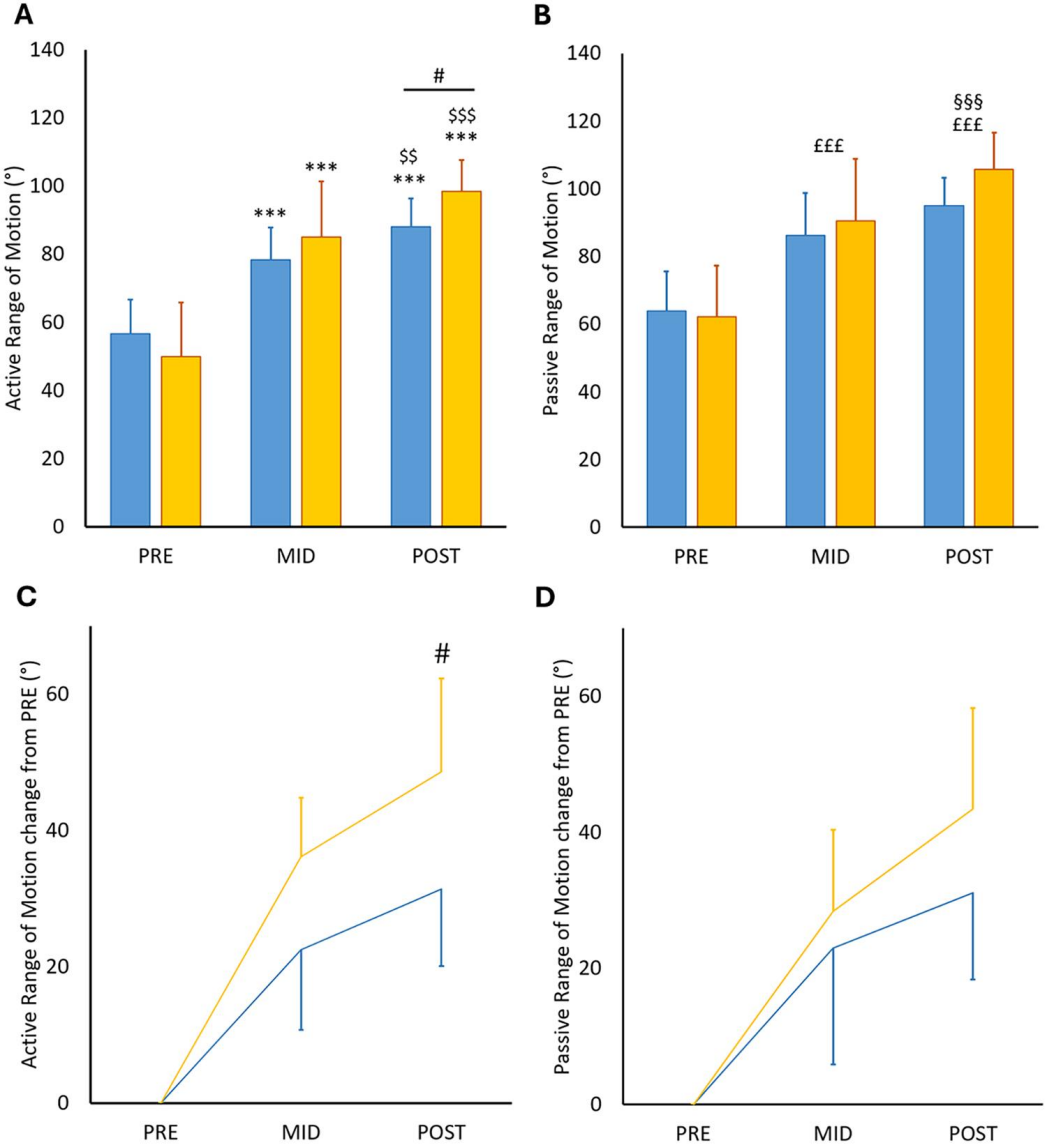
Knee range of motion measured at the end of the first session (PRE), fifth session (MID), and tenth rehabilitation session (POST) for the CPM group (blue bars and lines) and the CPM + FPS group (orange bars and lines). Data are presented as mean ± standard deviation (SD). **(A)** Active range of motion. **(B)** Passive range of motion. **(C)** Absolute change in active range of motion from PRE. **(D)** Absolute change in passive range of motion from PRE. ***p < 0.001 vs. respective PRE value for each group; ^$$^*p* < 0.01 and ^$$$^*p* < 0.001 vs. respective MID value for each group; ^£££^*p* < 0.001 vs. PRE for both groups (main effect of time); ^§§§^*p* < 0.001 vs. PRE for both groups (main effect of time); ^#^*p* < 0.05 between-group difference.

A significant group × time interaction (F(2,62) = 7.75; *p* = 0.001) and a significant main effect of time (F(2,62) = 160.44; *p* < 0.001) were observed for AROM. No significant between-group difference was observed at baseline (PRE) (*p* > 0.05; Table 1). AROM increased significantly in both groups throughout the two-week period (PRE to MID: +22.5 ±11.8°, 95% CI [15.0, 30.0], *p* < 0.001, ES = 2.22 for CPM and +36.1 ±8.7°, 95% CI [30.6, 41.6], *p* < 0.001, ES = 2.17 for CPM + FPS; MID to POST: +10.6 ± 6.1°, 95% CI [6.7, 14.5], *p* = 0.007, ES = 1.07 for CPM and +13.4 ±11.0°, 95% CI [6.5, 20.4], *p* < 0.001, ES = 1.01 for CPM + FPS; PRE to POST: +31.4 ± 11.3°, 95% CI [24.2, 38.6], *p* < 0.001, ES = 3.36 for CPM and +48.6 ±13.7°, 95% CI [39.9, 57.3], *p* < 0.001, ES = 3.73 for CPM + FPS; Figure 3A). At POST, AROM improvement was significantly greater in the CPM + FPS group compared with the CPM group (+48.6 ±13.7° vs. +31.4 ±11.3°, *p* = 0.036, ES = 1.37; Figure 3C). Absolute AROM changes throughout the rehabilitation program are illustrated in Figure 3C. Exploratory analyses were performed to evaluate achievement of commonly used rehabilitation milestones. After the first week (MID), 8 participants (67%) in the CPM + FPS group exceeded 90° in AROM, whereas 4 participants (33%) reached this threshold in the CPM group. At the end of the second week (POST), 2 participants in the CPM + FPS group achieved an AROM greater than 110°, while no participant in the CPM group reached this amplitude. These milestone analyses should be considered exploratory because the study was not specifically powered for categorical responder outcomes.

A trend toward a group × time interaction was observed for PROM (F(2,62) = 2.56; *p* = 0.083), along with a significant main effect of time (F(2,62) = 96.97; *p* < 0.001). No between-group differences were observed at baseline (*p* > 0.05; Table 1). Regardless of group, PROM increased significantly throughout the two-week period between PRE and MID (+25.7 ±14.7°, 95% CI [19.5, 31.9], *p* < 0.001, ES = 1.76; Figure 3B), MID and POST (+11.3 ±9.2°, 95% CI [7.4, 15.1], *p* < 0.001, ES = 0.89; Figure 3B), and PRE and POST (+37.3 ± 14.9°, 95% CI [31.0, 43.6], *p* < 0.001, ES = 3.07; Figure 3B). At POST, although no significant group × time interaction was observed, PROM gain was numerically greater in the CPM + FPS group compared with the CPM group (+43.5 ±14.8° vs. +31.1 ±12.7°; Figure 3D). Absolute PROM changes throughout the rehabilitation program are illustrated in Figure 3D. Because the overall group × time interaction for PROM did not reach statistical significance, the post-intervention between-group comparison should be considered exploratory and interpreted with caution Exploratory analyses were also performed and after the first week (MID), 9 of 12 participants (75%) in the CPM + FPS group achieved a PROM greater than 90°, compared with 6 of 12 participants (50%) in the CPM group. At the end of the second week (POST), only one participant in the CPM group achieved a PROM greater than 110°, whereas 5 participants in the CPM + FPS group exceeded this amplitude in PROM.

### 3.3 Knee extension deficit

No significant group × time interaction was observed for active (F(2,62) = 0.378; *p* = 0.687) and passive knee extension deficit (F(2,62) = 0.251; *p* = 0.779). A significant main effect of time was observed for both active (F(2,62) = 12.76; *p* < 0.001) and passive knee extension deficit (F(2,62) = 8.73; *p* < 0.001). Regardless of the group, active knee extension deficit was significantly improved between PRE and MID (+2.5 ± 4.7°, 95% CI [0.5, 4.4], *p* = 0.026, ES = 0.41), MID and POST (+2.1 ± 3.2°, 95% CI [0.7, 3.4], *p* = 0.033, ES = 0.44) and PRE and POST (+5.0 ± 5.6°, 95% CI [2.7, 7.3], *p* < 0.001, ES = 0.88) while passive knee extension deficit was significantly improved between PRE and MID (+2.4 ± 4.8°, 95% CI [0.3, 4.4], *p* = 0.041, ES = 0.45) and PRE and POST (+4.2 ± 5.8°, 95% CI [1.7, 6.7], *p* < 0.001, ES = 0.80).

### 3.4 Arthromotor amplitudes during rehabilitation

No significant group × time × sessions interaction was observed for arthromotor amplitudes (F(9,398) = 0.386; *p* = 0.942). No significant between-group difference was observed at the first session of the rehabilitation program. Significant main effects of time (F(1,398) = 236.69; *p* < 0.001) and session (F(9,398) = 97.49; *p* < 0.001) were observed. Regardless of group or session, arthromotor amplitudes increased significantly between the beginning and the end of each session (*p* < 0.001). Regardless of the group and the time of measurement (i.e., before or after the session), amplitudes increased progressively during the first three sessions (*p* = 0.018 and *p* < 0.001). From the third session, a significant increase in amplitudes was observed every two sessions throughout the two-week rehabilitation period (*p* = 0.030 to *p* < 0.001).

### 3.5 Pain scores and analgesic medication

**Figure 4:**
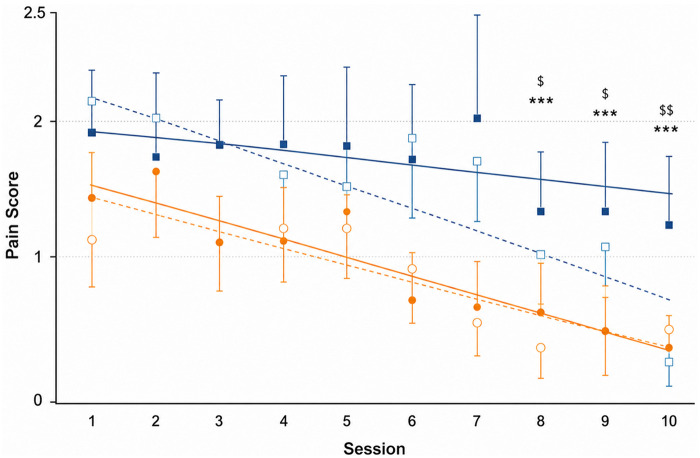
Pain scores reported before (empty symbols; dashed lines) and after the session (filled symbols; solid lines) for the CPM group (blue) and the CPM + FPS group (orange) throughout the ten rehabilitation sessions. Data are presented as mean ± standard error (SE). ^$^*p* < 0.05 and ^$$^*p* < 0.01 vs. session 1 (pooled values); ***p < 0.001 vs. session 2 (pooled values).

No significant group × time × sessions interaction was observed for pain scores (F(9,400) = 0.673; *p* = 0.733), and no between-group differences were observed at the first session. However, a significant main effect of session was observed for pain scores reported throughout the two-week period (F(9,400) = 4.834; *p* < 0.001). Regardless of the group and the time of measurement (i.e., before or after the session), pain scores recorded during sessions 8, 9 and 10 were significantly lower than those recorded during session 1 (*p* = 0.011, *p* = 0.017 and *p* = 0.007, respectively) and session 2 (*p* < 0.001, *p* = 0.001 and *p* < 0.001, respectively; Figure 4). No significant between-group difference in pain intensity was observed at any time point.

For analgesic medication use, no significant group × sessions interaction was observed (F(9,220) = 0.405; *p* = 0.932). However, a significant main effect of session was found (F(9,220) = 2.039; *p* = 0.036), with lower medication intake reported at session 10 compared with sessions 1–4 (*p* < 0.05).

The overall percentage of analgesic medication use was lower in the CPM + FPS group than in the CPM group across the rehabilitation period (66.0% vs. 87.7%). This descriptive result should not be interpreted as a direct measure of pain intensity reduction or superior analgesia.

## 4 Discussion

### 4.1 Principal findings

This preliminary randomized controlled trial investigated whether adding FPS to CPM during the first two weeks of rehabilitation after ACLR could improve early postoperative recovery of knee joint mobility. The main finding was that participants receiving CPM + FPS showed greater improvement in AROM than those receiving CPM alone. PROM also improved in both groups and was numerically greater in the CPM + FPS group at POST, without reaching statistical significance. Hence, PROM findings should be considered exploratory. Both groups improved knee extension deficit (in both active and passive conditions) and pain scores over time, with no significant between-group difference. Similarly, analgesic medication use decreased throughout the two weeks of rehabilitation without significant difference between groups. Nevertheless, the overall descriptive percentage of analgesic medication use was lower in the CPM + FPS group than in the CPM group. This secondary finding should be interpreted cautiously because analgesic use and pain intensity are related but distinct outcomes. Because no sham stimulation condition was included, the present study should be interpreted as estimating the pragmatic additive effect of FPS as delivered in clinical practice, rather than the specific physiological effect of proprioceptive stimulation independent of expectation, attention, novelty, or placebo-related influences.

### 4.2 Interpretation of range-of-motion findings

The greater improvement in AROM observed in the CPM + FPS group compared with CPM alone (+48.6° vs. +31.4°, respectively; ES = 1.37) suggests that adding synchronized proprioceptive stimulation to passive mobilization may facilitate active knee mobility during the early postoperative phase. This is clinically relevant because early restoration of knee flexion is a key rehabilitation target after ACL reconstruction and may influence progression toward later functional exercises. This finding is consistent with an earlier study reporting beneficial effects of FPS on knee joint mobility recovery following plaster cast removal, with increases of approximately 50° after 5 days and 90° after 30 days of rehabilitation (32). Similar improvements in joint mobility were also reported following wrist surgery for distal radial fracture, where a 7-day FPS intervention led to an increase of approximately 60° in wrist joint range of motion (33). In that study, FPS was applied on the non-operated wrist while both hands were maintained in palm-to-palm contact. Our findings are also consistent with a previous observation in patients with knee osteoarthritis, in whom an intervention combining CPM, local vibration and thermotherapy resulted in greater improvements in range of motion compared with a sham intervention (34). In the present study, PROM remained greater than AROM throughout the rehabilitation period, as expected during early postoperative recovery. This difference between passive and active mobility may reflect the influence of pain, postoperative apprehension, residual swelling, AMI, and reduced voluntary quadriceps activation. Although participants in the CPM + FPS group exhibited numerically greater improvements in PROM than the CPM group (+43.5° vs. +31.1°, respectively; ES = 0.90), the absence of a statistically significant group × time interaction indicates that the evidence supporting a differential treatment effect on passive mobility is weaker than that observed for AROM. Consequently, the PROM observations should be considered preliminary and require confirmation in larger studies specifically powered by this outcome. However, it is worth mentioning that the between-group difference in AROM and PROM (∼17° and ∼12°, respectively) is higher than the minimal detectable change values (i.e., 3.5°) reported for intrarater assessment in ACL participants (37). Then, from a clinical perspective, the magnitude of the AROM difference observed between groups may be meaningful because early restoration of knee flexion is a key objective of postoperative rehabilitation (17, 18). A greater proportion of participants in the CPM + FPS group achieved commonly targeted rehabilitation milestones, including 90° knee flexion after the first postoperative week and 110° flexion by the end of the second week. These milestone observations should be interpreted cautiously because the study was not powered for categorical responder analyses and universally accepted minimal clinically important differences for knee range of motion during the first postoperative weeks after ACLR are lacking.

### 4.3 Possible sensorimotor mechanisms

Several interacting mechanisms may explain why FPS combined with CPM was associated with greater AROM recovery. ACL injury and reconstruction can alter afferent input from the knee, while postoperative pain, swelling, inflammation, and joint effusion may contribute to AMI and impaired voluntary activation (3, 8). These factors can limit active motion even when passive mobility is improving. By stimulating muscle spindle afferents at the quadriceps and popliteal regions, FPS may provide additional proprioceptive input during passive movement and may help reinforce movement-related sensory information (38). In the CPM + FPS condition, vibration was synchronized with the passive movement generated by the arthromotor. This may have created a more coherent sensory experience than CPM alone by combining actual passive displacement with proprioceptive signals consistent with knee flexion and extension. Previous experimental studies have shown that local vibration can induce kinesthetic illusions and modulate corticospinal excitability (39–42). However, the present study did not directly assess kinesthetic illusion, proprioception, muscle activation, spinal reflex excitability, corticospinal excitability, cortical activity, or neuroplastic adaptations. Therefore, the current findings should be considered compatible with a sensorimotor facilitation hypothesis, but they do not demonstrate neuroplastic changes.

The potential influence of FPS on muscle function also deserves consideration. Previous studies have reported that local vibrations may acutely improve quadriceps function (30, 43) and muscle activation after ACLR (30). More importantly, local vibrations participate to strength recovery after ACLR (31, 44). In the present study, since muscle strength was not measured, it remains unknown whether the greater AROM improvement observed in the CPM + FPS group was accompanied by improvements in knee extensors strength or activation. Future studies should combine ROM assessment with direct measures of strength, electromyographic activity, and neurophysiological markers.

### 4.4 Pain, analgesic medication, and early rehabilitation

Pain intensity decreased over the two-week rehabilitation period in both groups, but no significant between-group difference was observed. This reduction in pain intensity over time is consistent with previous studies reporting analgesic benefits of CPM after ACLR (19, 20) as well as with the conclusions of a recent systematic review and meta-analysis indicating that CPM may contribute to pain reduction after knee surgery (25). However, contrary to our initial hypothesis and previous reports suggesting analgesic effects of vibration stimulation (16, 45), our results indicate that CPM + FPS did not produce a clearly superior effect on perceived pain intensity compared with CPM alone. Consequently, our results contrast with a previous finding showing greater pain reduction in patients with knee osteoarthritis following a four-week intervention combining CPM, local vibration, and thermotherapy compared with a sham intervention (34). Similarly, greater reductions in pain at rest and during movements were reported after a seven-day FPS intervention following surgery for distal radial fracture compared with a control group (33). A possible explanation for these divergent observations may be related to the relatively low baseline pain intensity observed in both groups in the present study. Baseline pain scores were low, suggesting that postoperative pain was partially controlled before study enrolment. Consequently, the potential for further reductions in pain intensity may have been limited, creating a floor effect that reduced the ability to detect between-group differences.

Although a lower percentage of analgesic medication use during the rehabilitation period was noted for the CPM + FPS group in comparison with the CPM group (66% vs. 87.7%, respectively), this descriptive difference should not be considered direct evidence of superior analgesia. Medication intake can be influenced by several factors, including individual pain coping strategies, expectations regarding recovery, personal attitudes toward medication, clinician advice, prescribing practices, and the timing of pain relative to rehabilitation sessions. In addition, because medication use was recorded only as a binary daily variable without dosage, timing, or medication type information, the present study cannot determine whether participants used less medication overall or simply reported fewer days of intake. Additional studies specifically designed to evaluate pain-management outcomes are needed to clarify the clinical significance of this observation and to ascertain the analgesic effect of vibration stimulation (15, 16) after ACLR.

Pain may also influence sensorimotor recovery through mechanisms extending beyond subjective discomfort. Pain, swelling, inflammation, and joint effusion can contribute to AMI, altered proprioceptive processing, and changes in motor control. Appropriate pain management may facilitate participation in rehabilitation and reduce movement avoidance. Conversely, excessive analgesia could theoretically mask clinically relevant symptoms and interfere with pain-guided load management. Because these mechanisms were not directly assessed, the lower percentage of analgesic medication use in the CPM + FPS group should be interpreted as an exploratory secondary finding rather than definitive evidence of improved pain control.

### 4.5 Clinical implications

The present findings suggest that incorporating FPS during CPM sessions may represent a feasible adjunct within a broader early rehabilitation program following ACLR. FPS can be applied during passive mobilization without requiring active participant participation, which may be relevant during the first postoperative days when pain, swelling, apprehension, and limited voluntary activation can restrict active exercise participation. However, CPM + FPS should not be interpreted as a substitute for comprehensive physiotherapy. In the present protocol, CPM + FPS or CPM represented only the first 30 min of each three-hour session. The remaining intervention included lower-limb strengthening, gait training using an anti-gravity treadmill, core stability exercises, manual physiotherapy, compressive cryotherapy and pressotherapy. These components were intended to address neuromuscular control, multiplanar stabilization, and functional movement patterns. Thus, the results of the present study should be interpreted within the context of a broader standardized rehabilitation program, not as the isolated effect of CPM or FPS alone.

### 4.6 Strengths and limitations

A strength of the present study is that it investigated a clinically applicable intervention during the acute postoperative phase after ACLR, using a standardized rehabilitation protocol delivered in a real clinical setting. The repeated assessment of AROM, PROM, extension deficits, pain, and analgesic use provides a detailed description of early recovery during the first two postoperative weeks.

Several limitations should be considered when interpreting the present findings. First, the sample size was modest. Although the study was designed around the AROM recovery, it may have been underpowered to detect smaller effects on secondary outcomes such as pain intensity, milestone achievement, or analgesic medication use. Second, no sham stimulation condition was included. Therefore, expectation effects, attention effects, novelty effects, and placebo-related influences cannot be excluded, although a previous study demonstrated the beneficial effects of local vibration over a sham condition (34). This limitation is particularly relevant for outcomes potentially influenced by perception and behavior, including pain, perceived movement amplitude, willingness to increase the CPM range of motion, and active engagement during subsequent rehabilitation exercises. Third, blinding of participants and treating physiotherapists was not feasible because the FPS intervention produced perceptible sensory stimulation. Although outcome assessors were blinded to group allocation, no formal evaluation of blinding success was performed. Fourth, detailed screening logs were not prospectively maintained and the number of participants assessed for eligibility, excluded before enrolment, or declining to participate could not be fully reconstructed retrospectively.

Additional limitations relate to outcome measurement and clinical confounding. The study cannot determine whether the observed differences reflected reduced analgesic requirements, differences in prescribing practices, or other factors influencing medication use. Other potentially relevant clinical variables, including preoperative range of motion, prehabilitation status, preoperative activity level, time from injury to surgery, and objective measures of joint effusion or swelling, were not systematically collected. Although randomization was intended to balance both measured and unmeasured factors between groups, residual confounding cannot be completely excluded. Finally, although participants were monitored throughout the rehabilitation period and no adverse events were reported, no formal adverse-event adjudication process was implemented, so minor events not affecting rehabilitation progression may have been underreported.

### 4.7 Future directions

Future studies should include larger samples and include direct assessments of muscle strength, quadriceps activation, swelling, pain-related fear, proprioception, and neurophysiological markers. Such measures would help determine whether FPS primarily improves joint mobility through enhanced proprioceptive input, reduced protective inhibition, improved tolerance to movement, or other sensorimotor mechanisms. In addition, future trials should consider sham stimulation and standard-care control groups to better isolate the specific effects of FPS and CPM.

## 5 Conclusion

Within the context of a standardized early rehabilitation program following ACLR, the addition of FPS to CPM was associated with greater improvements in active knee range of motion during the first two postoperative weeks compared with CPM alone. Although passive range of motion tended to improve to a greater extent in the CPM + FPS group, the evidence supporting this finding was less robust and should be interpreted cautiously. Although reduced during the two weeks program, no significant between-group difference was observed for pain intensity. A lower descriptive percentage of analgesic medication use was noted for the CPM + FPS group. However, because medication use was assessed as a binary outcome and pain intensity did not differ significantly between groups, the clinical significance of this finding remains uncertain. These preliminary findings support the potential value of FPS as a pragmatic adjunct to CPM during early postoperative rehabilitation aimed at improving knee mobility. However, because no sham stimulation condition was included and muscle strength, proprioception, and neurophysiological outcomes were not directly assessed, the specific physiological contribution and mechanisms of FPS remain to be established in larger randomized controlled trials to confirm these preliminary observations.

## Data Availability

The original contributions presented in the study are included in the article/Supplementary Material, further inquiries can be directed to the corresponding author.
